# Chemoresistance in Pancreatic Cancer

**DOI:** 10.3390/ijms20184504

**Published:** 2019-09-11

**Authors:** Siyuan Zeng, Marina Pöttler, Bin Lan, Robert Grützmann, Christian Pilarsky, Hai Yang

**Affiliations:** 1 Department of Surgery, Universitätsklinikum Erlangen, Krankenhausstraße 12, 91054 Erlangen, Germany; siyuan.zeng365@gmail.com (S.Z.); bin.lan1991@gmail.com (B.L.); Robert.Gruetzmann@uk-erlangen.de (R.G.); christian.pilarsky@uk-erlangen.de (C.P.); 2 Department of Otorhinolaryngology, Head and Neck Surgery, Universitätsklinikum Erlangen, Glückstraße 10a, 91054 Erlangen, Germany; Marina.Poettler@uk-erlangen.de

**Keywords:** pancreatic cancer, PDAC, FOLFIRINOX, gemcitabine, chemoresistance

## Abstract

Pancreatic ductal adenocarcinoma (PDAC), generally known as pancreatic cancer (PC), ranks the fourth leading cause of cancer-related deaths in the western world. While the incidence of pancreatic cancer is displaying a rising tendency every year, the mortality rate has not decreased significantly because of late diagnosis, early metastasis, and limited reaction to chemotherapy or radiotherapy. Adjuvant chemotherapy after surgical resection is typically the preferred option to treat early pancreatic cancer. Although 5-fluorouracil/leucovorin with irinotecan and oxaliplatin (FOLFIRINOX) and gemcitabine/nab-paclitaxel can profoundly improve the prognosis of advanced pancreatic cancer, the development of chemoresistance still leads to poor clinical outcomes. Chemoresistance is multifactorial as a result of the interaction among pancreatic cancer cells, cancer stem cells, and the tumor microenvironment. Nevertheless, more pancreatic cancer patients will benefit from precision treatment and targeted drugs. Therefore, we outline new perspectives for enhancing the efficacy of gemcitabine after reviewing the related factors of gemcitabine metabolism, mechanism of action, and chemoresistance.

## 1. Introduction

Ranking as the fourth leading cause of cancer-related mortality in western countries, pancreatic cancer is predicted to become the second leading cause of cancer-related death after lung cancer by 2030 [1]. Pancreatic cancer remains a deadly malignancy, with few symptoms showing before the disease reaches its advanced stage [2]. According to reports, globally, 458,918 new cases of pancreatic cancer were found in 2018, causing 432,242 deaths [3]. Despite progress in the techniques of detecting and managing pancreatic cancer, the five-year survival rate only reaches to about 9% [3,4].

Approximately 90% of pancreatic malignancies are pancreatic ductal adenocarcinomas (PDACs). The primary risk factors related to pancreatic cancer involve smoking, alcohol abuse, diabetes mellitus, obesity, aging, family history, and genetic factors [3,5]. The main factors like low rate of early detection, rapid progression, the development of drug resistance, and lack of proper therapy lead to a poor prognosis of PC. Most primary pancreatic cancer patients lack corresponding clinical symptoms, and the existing screening biomarkers, such as CA19-9, have shown low sensitivity and specificity in diagnosing PC. [6,7]

## 2. Current Treatment Options of Pancreatic Cancer and Limitations

The current standard of care for resectable pancreatic ductal adenocarcinoma (PDAC) is surgery first followed by adjuvant chemotherapy [8]. However, because of a lack of appropriate early detection and screening methods, most pancreatic cancer patients are diagnosed with advanced or metastatic disease. Only 15% to 20% patients with pancreatic cancer are surgically treated when they are diagnosed, even among patients who have received surgical resections, because most patients will suffer from disease recurrence within a year [9]. The fundamental reasons for these frustrating outcomes are related to poor therapeutic results as an adjuvant surgical treatment, undetected micrometastasis, and the development of chemical resistance.

In the last decade, gemcitabine has been considered as the standard treatment and been widely utilized as a first-line drug for advanced pancreatic cancer [10]. The median overall survival rate (5.65 vs. 4.41 months) and one-year survival rate (18% vs. 2%) for gemcitabine-treated patients was significantly improved compared with 5-fluorouracil (5-FU) [10]. Notably, the clinical efficacy response of gemcitabine showed satisfying outcomes, approximately five times that of 5-FU (23.8% vs. 4.8%). Two combination regimens for metastatic pancreatic cancer turned out to be gold standards in recent years: 5-fluorouracil (5-FU)/leucovorin with irinotecan and oxaliplatin (FOLFIRINOX) [11], and gemcitabine with nab-paclitaxel since 2011 [12,13,14]. Several trials have been conducted to test the efficacy of different drugs, either alone or in combination with gemcitabine, and eventually achieved modest success. A recent study showed that adjuvant therapy with a modified FOLFIRINOX (oxaliplatin, irinotecan, leucovorin, and fluorouracil) regimen brought a profoundly longer survival than gemcitabine among resected pancreatic cancer patients [15]. The median disease-free survival (DFS) was significantly higher in the modified-FOLFIRINOX group compared with the gemcitabine group (21.6 vs. 12.8 months). The median overall survival (OS) in the modified-FOLFIRINOX group was 54.4 months and 35.0 months in the gemcitabine group.

## 3. Chemoresistance in Pancreatic Cancer

Although gemcitabine and other therapeutic drugs are effective among patients with advanced and metastatic PC, development of chemoresistance to gemcitabine severely limits the effectiveness of this chemotherapy. It is undeniable that pancreatic cancer cells are more resistant to gemcitabine than other chemotherapeutic drugs. Since research on the effects of other drugs is still in its infancy, most studies on chemoresistance in advanced PC have focused on gemcitabine. The underlying mechanism for the development of gemcitabine resistance remains unclear. Various transcription factors, such as enzymes and signaling pathways, involved in nucleoside metabolism are more or less included in the development of chemoresistance to gemcitabine [16,17,18].

## 4. Mechanism of Action of Gemcitabine

**Figure 1:**
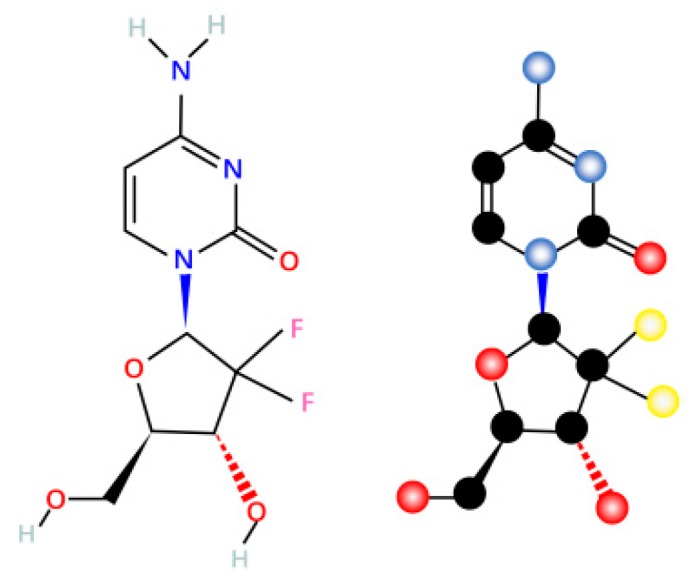
Gemcitabine molecular formula and structure.

Gemcitabine, also known as dFdC: 2′,2′-difluoro-2′-deoxycytidine (dFdC) (Figure 1), is a deoxycytidine nucleoside analog whose function is mainly dependent on several inhibitions of DNA synthesis [19], thereby inhibiting proliferation and blocking the cell cycle process at the G1/S phase boundary [20].

Gemcitabine requires intracellular phosphorylation to function after uptake, and its activation and transport are governed by various enzymes [21]. Therefore, nucleoside transport activity is a prerequisite for inhibiting cell growth. This hydrophilic molecule can be transported into cells through a variety of human nucleoside transporters (NTs), including sodium-dependent (concentrative nucleoside transporter hCNTs) and sodium-independent (equilibrative nucleoside transporter hENTs) transporters [22]. The intracellular uptake of dFdC is mainly mediated by hENT1, while the hCNTs family mediates the unidirectional transport of nucleosides into cells. [19].

**Figure 2:**
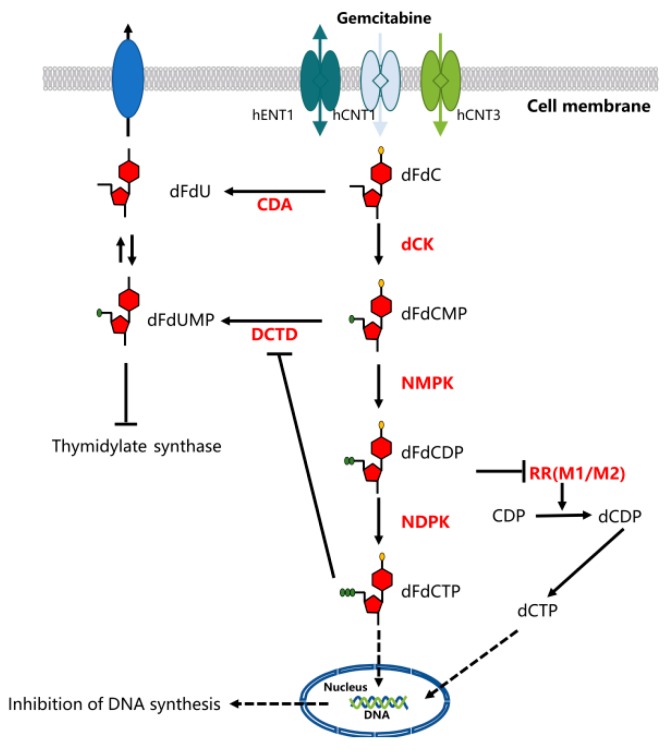
Mechanism of action of gemcitabine. Gemcitabine activation and transport are governed by various enzymes. Nucleoside transporters include the human concentrative nucleoside transporters (hCNTs) and human equilibrative nucleoside transporters (hENTs). After gemcitabine enters the cell membrane, deoxycytidine kinase (dCK) is the first phosphorylated rate-limiting enzyme and phosphorylates gemcitabine to gemcitabine monophosphate (dFdCMP). Subsequently, complex intracellular converts to nucleotide gemcitabine diphosphate (dFdCDP) and triphosphate (dFdCTP). Gemcitabine metabolite dFdCTP inhibits ribonucleoside reductase (RR), an enzyme that regulates DNA biosynthesis, by controlling the formation of nucleoside triphosphates (NTPs). RR transforms CDP into dCDP, and its inhibitory effect leads to the decreased concentration of competitive dCTP pool cells required for DNA synthesis, thus promoting the binding of dFdCTP to DNA.

The cytotoxic activity of gemcitabine may be the result of multiple effects of DNA synthesis. Gemcitabine was phosphorylated into gemcitabine monophosphate (dFdCMP) by deoxycytidine kinase (dCK) after an influx of nucleoside transporters into cell membranes, which underwent complex intracellular transformation to gemcitabine diphosphate (dFdCDP) and triphosphate (dFdCTP), responsible for its cytotoxicity. DNA polymerase can be inhibited by competition with dFdCTP and deoxycytidine triphosphate (dCTP). The effective inhibitor of ribonucleoside reductase, dFdCDP, can lead to depletion of the deoxyribonucleotide pools, which are necessary for DNA synthesis, thereby further enhancing the action of dFdCTP. The dFdCTP is incorporated into the DNA, and after incorporation of another nucleotide, the DNA strand is terminated. This process of “masked chain termination” can hide dFdCTP from DNA repair enzymes and lock gemcitabine into DNA, which leads to the failure of normal DNA repair mechanisms. [19]. Gemcitabine also possesses a unique mechanism to enhance its own activation, termed “self-potentiation”. This “self-potentiation” prolongs the maintenance of high intracellular concentrations of gemcitabine metabolites and increase, which prolongs the maintenance of high intracellular concentrations of gemcitabine metabolites and increases the probability of successful incorporation of gemcitabine into nucleic acids, mainly DNA, by reducing competing natural metabolites [21]. Gemcitabine metabolite dFdCDP potently inhibits ribonucleotide reductase (RR), resulting in a decrease of competing deoxyribonucleotide pools necessary for DNA synthesis [21,23]. Additionally, the intracellular decrease of dFdCTP and dCTP inhibited the inactivation of dCTD to dFdCMP, and the activity required an adequate level of active dCTP [19,21] (Figure 2).

## 5. Gemcitabine Resistance Mechanisms

Chemoresistance can be divided into two categories [24], which may be an initial property but can also be obtained in the course of drug treatment [25]. Certain drug-resistant mechanisms have been depicted, relating to drug transporters, activating and inactivating enzymes and its targets. The transport, activation, and metabolism of gemcitabine are involved and regulated by a variety of enzymes, and the formation of drug resistance is regulated by various factors, such as the tumor microenvironment, epithelial–mesenchymal transition, and microRNA. We will mainly discuss the role and mechanism of these factors in gemcitabine chemoresistance.

### 5.1. Nucleoside Transporters

Gemcitabine is known to require a nucleoside transporter (NT) to pass through the plasma membrane and display pharmacological activity [19,22]. Restriction of intracellular uptake of gemcitabine by reducing hENT1 expression is an established mechanism of drug resistance [22,26]. Studies have shown that the survival rates of patients with a lower expression of nucleoside transporters were significantly decreased [27,28,29,30,31]. Since the high expression of hENT1 is related to longer OS and DFS in pancreatic cancer patients, the expression level of hENT1 can be used as a prognostic indicator in resected pancreatic cancer patients who received gemcitabine adjuvant chemotherapy [29,30]. hENT1 activity is a prerequisite for in vitro growth inhibition and a crucial determinant of its sensitivity to gemcitabine, so the lack of hENT1 may lead to significant drug resistance of gemcitabine [26]. hENTs transported GEM with different characteristics, and hENT1 could uptake GEM with high affinity, but its capacity was low, while hENT2 transported gemcitabine with low affinity and high capacity. [32,33]. The recent study indicated that liver extraction of GEM is mainly mediated by low affinity hENT2, and that hENT2-mediated uptake is not fully saturated, even at high drug concentrations. Recent data indicate that the expression of a member of the concentrative nucleoside transporters, hCNT1, is constantly decreased in PC cell lines compared with normal pancreatic ductal epithelial cells, and can induce cell cycle arrest and increase cell apoptosis through non-apoptotic mechanisms and inhibit cell migration [34,35]. Moreover, it is worth nothing that hCNT1 protein repair can suppress tumor growth in a mouse model of pancreatic cancer as well. Drug inhibition of degradation of hCNT1 can moderately increase the transport of gemcitabine and improve the drug effect of gemcitabine [36].

### 5.2. Nucleoside Enzymes

Once gemcitabine is transported into cells by NTs, deoxycytidine kinase will become the main rate-limiting enzyme for intracellular activation and metabolism of gemcitabine. The inactivation of dCK is one of the key mechanisms of gemcitabine resistance. The significant correlation between gemcitabine sensitivity and dCK activity has been confirmed in human and mouse xenografts. The obtained resistance of PDAC cell lines to gemcitabine suggests that dCK is often inactivated [37]. Knockdown of the *dCK* gene often leads to gemcitabine resistance [38], while overexpression of *dCK* can restore the chemosensitivity of gemcitabine in gemcitabine-resistant cell lines [39,40]. A large number of clinical studies have shown that the dCK expression level in pancreatic cancer tissue is a reliable prognostic indicator of PFS, so dCK may be the best biomarker of dFdC sensitivity for PC patients treated with gemcitabin [41,42].

Hu antigen R (HuR), an RNA-binding protein that post-transcriptionally regulates dCK, is also related to gemcitabine efficacy [43,44,45]. HuR-overexpressing cancer cells are about 30 times more sensitive to gemcitabine therapy. HuR is strongly associated with dCK mRNA in pancreatic cancer cells. Gemcitabine treated pancreatic cancer cells can enrich dCK mRNA and improve cytoplasmic HuR levels. Therefore, the overexpression of HuR will increase, and the silence of HuR will reduce the expression of dCK protein, thus producing the corresponding gemcitabine response in pancreatic cancer [44].

Another key enzyme involved in gemcitabine resistance is ribonucleotide reductase (RR). RR is a rate-limiting enzyme in the DNA synthesis pathway. It is primarily responsible for turning ribonucleotides into dNTPs, which is critical for DNA assembly and repair. RR is composed of two subunits, M1 and M2. Inhibition of RR induced by dFdCDP is the most significant mechanism for the enhancement of gemcitabine activity [20,21]. A recent meta-analysis revealed that patients with high RRM1 expression had largely poorer OS and DFS than those with low RRM1 expression [46,47], suggesting that the expression of RRM1 is an indicator of poor survival in patients with pancreatic cancer accepting gemcitabine chemotherapy [46]. Experiments confirmed that overexpression of RRM1 and RRM2 proteins of the pancreatic cancer cell line can achieve stable genetic gemcitabine resistance [48,49]. Increased RR activity increased the size of the dNTP library and competitively inhibited dFdCTP incorporation into DNA. The extended dNTP pool further downregulated DCK activity and reduced gemcitabine phosphorylation through the negative feedback pathway. Excessive dCTP may be a positive feedback mechanism of dCMP deaminase, leading to increased gemcitabine metabolism [49,50]. Therefore, RR may be an effective target for the treatment of acquired drug resistance in pancreatic cancer [51].

### 5.3. Epithelial–Mesenchymal Transition

Epithelial–mesenchymal transition (EMT) is a phase of phenotypic change in tumor cells that favors a higher aggressive mesenchymal phenotype. This process is generally accompanied by morphological changes in cancer cells and changes in genome and protein levels. However, the possible role of mesenchymal transcription factors in chemotherapeutic resistance has received more attention recently. The EMT process is mediated by a variety of key genes and cellular signaling pathways. There is a strong negative correlation between E-cadherin and Zeb-1, and it is closely related to chemoresistance. The presence of Zeb-1 and other modulators of EMT maintains drug resistance in human pancreatic cancer cells. When the Zeb-1 gene was silenced, the protein expression level of E-cadherin was upregulated, and drug sensitivity was restored by improving the expression of the epithelial markers, EVA1 and MAL2 [52]. Snail and Twist were two key transcription factors responsible for EMT. Inhibition of the EMT process resulted in improved expression of nucleoside transporters in tumors, thus enhancing sensitivity to gemcitabine therapy [53]. Signaling pathways, such as Notch, tumor necrosis factor alpha (TNFα), transforming growth factor beta (TGF-β), and hypoxia-inducible factor-1 alpha (HIF1α), are involved in the induction of EMT in pancreatic cancer cells [54]. Nevertheless, ENT1 and CNT3 are frequently upregulated in KPC mice models with deleted Snail or Twist, and the related signaling pathway between GEM resistance and EMT remains unclear. Importantly, independent EMT pathways, such as MAPK activation, transporters, and gemcitabine metabolism enzymes, can also cause GEM resistance, thus increasing the complexity of this process [55]. Further research indicated that USP27X is required for TGFβ-induced Snail1 expression and is upregulated by TGFβ during EMT. Depletion of USP27X effectively attenuates Snail1-dependent cell invasion and migration, as well as metastasis formation, and prevents TGFβ-induced EMT and fibroblast activation. Inhibition of USP27X is proposed as a target for Snail1-dependent tumor invasion and chemoresistance [56].

The EMT phenotype is not only related to cancer cell proliferation but also participates in the formation of gemcitabine resistance. Arumugam and colleagues used a panel of PDAC cell lines with different sensitivities to gemcitabine and 5-fluorouracil, and based on global gene expression profiles, confirmed that drug-resistant cells contain many features consistent with EMT. Reversing EMT by silencing of Zeb-1 not only restored the expression of typical epithelial marker genes but also increased the sensitivity of the cells to therapeutic agents, suggesting that Zeb-1 and other EMT regulatory factors maintain drug resistance in human pancreatic cancer cells [52]. One morphological feature recently suggested as a possible reliable marker of poor prognosis in PDAC is tumor budding (TB). TB is thought to morphologically reflect the process of EMT, which allows tumor epithelial cells to acquire more mesenchymal phenotypes, thereby increasing their ability to migrate and invade, helping them to develop resistance to chemotherapy drugs [57,58,59]. Hematoxylin-eosin staining, immunohistochemistry, and other histological examinations can effectively evaluate tumor budding [58,59,60]. Currently, a large number of studies have indicated that tumor budding is significantly correlated with EMT, due to an increased expression of mesenchymal markers, such as vimentin [59], ZEB1, and ZEB2 [57], in TB cells, while showing reduced or focal expression of epithelial markers, such as E-cadherin and cytokeratin. A recent meta-analysis of the prognostic role of high-grade tumor germination in PDAC shows that EMT is a central process in determining the presence of TB in PDAC, and that high-grade TB has potential clinical significance in stratifying PDAC prognosis [58]. Furthermore, the association of tumor budding with vimentin expression supported the idea that EMT is a key process in PDAC responsible for progression and drug resistance [59]. Although there is a significant correlation between budding and EMT, and EMT is involved in the formation of drug-resistance, the ability to predict potential chemoresistance through histological examination of TB still requires extensive clinical practice and further research.

### 5.4. Microenvironmental Factors

The tumor microenvironment is receiving great attention due to its complex cell type and intricate cell signaling pathway, which plays a pivotal role in the development of cancer. Interstitial tissues surround pancreatic cancer, including pancreatic stellate cells (PSCs), fibroblasts, inflammatory cells, endothelial cells, nerve cells, and other components, together constituting the tumor microenvironment [61,62]. Remarkably, it also makes an important contribution to the chemotherapy resistance process in PC [63,64,65,66]. PSCs are the main components of tumor stroma, which are inactive in normal tissue but activated by secreted factors, such as TNFα, TGF-β, and interleukins 1,2,10 (IL1, IL2, IL10) [61]. These activated PSCs form a dense matrix around them by secreting extracellular matrix (ECM) proteins, including collagen, fibronectin, and laminin. In vitro co-culture experiments confirmed that PSCs can increase Hes1 expression through the Notch signaling pathway, thereby enhancing chemoresistance to GEM. PSC-induced chemoresistance can be effectively reversed when using Notch signaling pathway inhibitors or silencing the expression of the *Hes1* gene [64]. The formation of the tumor microenvironment is involved in many factors. Recent studies have shown that the periostin is only overexpressed in the PDAC stroma and the PSCs, which not only drives the carcinogenic process but also has the ability to confer GEM resistance to pancreatic cancer cells [66]. PSCs also promote chemoresistance to gemcitabine by paracrine SDF-1α/CXCR4 signaling-induced activation of the intracellular FAK-AKT and ERK1/2 signaling pathways and a subsequent IL-6 autocrine loop in cancer cells [65]. The Hedgehog (Hh) pathway is a signaling cascade that plays a crucial role in many processes, including embryonic development and tissue homeostasis [67]. In addition, emerging evidence suggests that abnormal activation of Hh is associated with tumor transformation, malignancy, and drug resistance [68,69,70]. At the molecular level, Hh signaling has been shown to drive cancer progression by modulating cancer cell proliferation, malignancy, metastasis, and expansion of cancer stem cells (CSCs) [67,71,72,73]. The expression of sonic hedgehog (SHH) influences the motility and differentiation of fibroblasts and PSCs and then affects tumor growth by promoting the formation of desmoplasia in PC [74]. Studies have shown that activation of Hh could increase the matrix in the microenvironment of pancreatic cancer, and inhibition of Hh induced a decrease of α-smooth muscle actin. A reduction in the matrix could increase the mean vessel density (MVD), thereby improving blood perfusion and drug delivery [75]. After cyclopamine-mediated Hh blockade, a decrease in proliferation of PDAC was observed and chemoresistance to gemcitabine was reversed, resulting in decreased expression of ATP binding cassette subfamily G member 2 (ABCG2) in PDAC [76]. Furthermore, in a mouse model, studies have shown that by inhibiting the Hedgehog cell signaling pathway, depletion of tumor-associated stromal tissue leads to a transient increase in intravascular tumor density and concentration of gemcitabine, making the disease temporarily stable [75].

Cancer-related fibroblasts (CAFs) and pancreatic cancer are related to various facets of proliferation, migration, and chemotherapeutic resistance. CAFs are produced by bone marrow-derived mesenchymal stem cells (MSCs), PSCs, and quiescent resident fibroblasts through a variety of pathways of activation, including EMT [77]. Chemoresistance can be reduced through inhibition of the mTOR/4E-BP1 pathway, which is highly active in alpha-smooth muscle actin (SMA)-positive CAFs. The SOM230 analogue (Pasireotide) is a recently discovered inhibitor of the mTOR/4E-BP1 pathway that activates the sst1 receptor and inhibits synthesis of the secreted protein IL-6. The combination of SOM230 and gemcitabine in mouse xenografts can lead to a decrease of tumor growth and chemoresistance, decrease of fibrosis, and increase of cell death induced by gemcitabine [78]. CAFs also robustly express activated IRAK4 and NFκB. IRAK4 expression in CAFs, promotes NFκB activity and the fibrosis of tumors, and supported the proliferation, survival, and chemoresistance of PDAC cells. IL1β acts as a key cytokine that activates IRAK4 in CAFs. Targeting of IRAK4 or IL1β was less fibrotic and more sensitive to gemcitabine in PC [79].

### 5.5. Impact of Other Relevant Factors

The formation of gemcitabine resistance is affected by many factors, and its drug resistance process is a multi-level linkage and cumbersome process. MicroRNAs (miRNAs) are a class of small non-coding RNAs of 19 to 25 nucleotides that negatively regulate genes at the post-transcriptional level [80,81]. Lacking the ability to encode a protein, miRNAs bind mainly to the 3′ or 5′ untranslated region of their target mRNAs that are imperfectly complementary. The consequences of miRNA binding are that the bound mRNA is either silenced or degraded, resulting in reduced levels of the protein encoded by the mRNA [82]. An increasing number of publications in recent years correlate miRNA expression in PC with resistance or sensitivity towards chemotherapeutic targets [81,83,84,85]. Treatment with gemcitabine led to the upregulation and downregulation of various miRNAs, and the expression of related proteins is mediated through different signaling pathways, resulting in chemoresistance in PC. The overexpression of microRNA-210 was toxic to gemcitabine-resistant cells and enhanced gemcitabine sensitivity and also reduced ABCC5 mRNA levels and inhibited the luciferase reporter gene expressing the ABCC5 3′UTR [86]. The inhibition of miR-101-3p in gemcitabine-resistant PC cells is a key regulator of RRM1 upregulation. Gemcitabine-resistant cells had low levels of miR-101-3p and the lipofection of miR-101-3p mimics led to inhibition of RRM1 expression and restored gemcitabine sensitivity [87]. Similarly, the downregulation of miRNA-124 via the polypyrimidine tract binding protein (PTBP1) and pyruvate kinase pathway also increased the resistance of gemcitabine [88]. Treatment with gemcitabine also led to the upregulation of miRNA-17-5p via the phosphatase and tensin homolog (PTEN) pathway [89], miRNA-21 via the PTEN/Akt pathway [90], and miRNA-203 via the activation of salt-inducible kinase (SLK1) [91]. The upregulation of these miRNAs due to gemcitabine treatment resulted in chemoresistance. Schlafen 11 (SLFN11) is widely expressed in a variety of cancer cells, and its expression levels are related to the sensitivity of cells to chemotherapeutic drugs, especially DNA-damaging agents (DDAs) [92,93]. All these drugs damage DNA during the S-phase, activating the intra-S-phase checkpoint and inactivating replication fork slowing [94]. Studies have shown that SLFN11 is a DNA damage response protein. When DNA is damaged, SLFN11 is recruited to the DNA damage site, co-locates with single-stranded DNA (ssDNA) binding protein replication protein A (RPA) [95], and SLFN11 then inhibits checkpoint maintenance and homologous recombination repair by promoting the destabilization of the RPA–ssDNA complex, thereby sensing cancer cell lines with high SLFN11 to DDAs [96]. Using cells with high or low endogenous SLFN11 expression or cells subjected to siRNA-mediated silencing of SLFN11, researchers showed SLFN11 to be causative in determining cell death and cell cycle arrest in response to DDAs in cancer cells. It is worth mentioning that SLFN11 is inactivated in about 50% of cancer cell lines and most tumors, and cells with lower expression of SLFN11 are highly sensitive to chemotherapeutic drugs [97,98]. These results demonstrate the importance of SLFN11 in response to DDAs and suggest the importance of testing SLFN11 expression as a predictive biomarker, with a potential benefit for a large number of patients with multiple cancer types treated with DDAs. In addition, methylation may be the main reason for the decreased expression level of SLFN11 [98,99], as methylation of SLFN11 leads to decreased gene expression and loss of function in tumor cells [99]. The sensitivity of cancer cells to chemotherapeutic drugs can be restored by inhibiting SLFN11 methylation in vivo. Overall, the SLFN11 expression level and its degree of methylation can be used as predictive biomarkers of the therapeutic effects of chemotherapy drugs [100].

## 6. Potential Ways to Improve Gemcitabine Uptake and Efficacy

Gemcitabine demonstrates a low therapeutic efficacy because of the poor membrane permeability and unstable metabolism. Table 1 summarizes the recent research of potential ways to enhance the efficacy of gemcitabine. Gemcitabine depends on NTs for intracellular uptake; NTs are important in effective treatment with gemcitabine. Pretreatment with thymidylate synthase (TS) inhibitors can enhance hENT1 expression and increase the therapeutic effect of gemcitabine [22]. It has been shown that transmembrane glycoprotein mucin 4 (MUC4) suppresses hCNT1 expression through the NF-κB pathway, while inhibition of MUC4 leads to enhanced gemcitabine sensitivity [101]. Silencing of the oncogenic receptor, the membrane partner of MUC4, leads to improved gemcitabine sensitivity by upregulation of hCNT1 and hCNT3 expression [102].

Currently, the solution to gemcitabine resistance in pancreatic cancer is to bypass the NTs and dCK through different mechanisms or drug modifications, thereby enhancing the transport and phosphorylation of gemcitabine.

NEO6002 is a modified prodrug that binds cardiolipin to the GEM. The mouse tumor xenograft model confirms that NEO6002 enters the cell-independent activity of NTs. NEO6002 exerts higher activity and lower toxic side effects in cells than GEM alone [103]. Another lipophilic prodrug, gemcitabine-elaidic acid conjugate CP-4126, has been shown to be transfused into cancer cells independent of hENT1 levels [104,105]. CP-4126 has a wide range of antiproliferative effects, and its safety and efficacy have been demonstrated in vitro and in various human cancer models, including pancreatic cancer [104]. CP-4126 and GEM are equally effective in chemoresistant cancer cell lines and multiple xenotransplantation models. However, both of them are ineffective in cells lacking dCK activity [104]. Apart from their independence of transporter uptake, CP-4126 appears to accumulate in cell vesicles, which make drugs stay in cells for longer periods. In phase I and pharmacokinetics studies, CP-4126 was comparable to gemcitabine in toxicity and had good tolerance [106,107]. However, it is horrifying that in a large randomized phase II study, long-term survival analysis confirmed that the survival rate of patients using CP-4126 was not superior to gemcitabine in patients with low expression of hENT1 in metastatic PDAC. Furthermore, the response to CP-4126 was not observed by the level change of hENT1, and the correlation between hENT1 levels and gemcitabine sensitivity was not shown [108]. As described above, when gemcitabine is transported into cells, dCK is considered to be the key rate-limiting enzyme required for the phosphorylation of gemcitabine into a monophosphate form. Therefore, the phosphorylation of gemcitabine bypasses dck by prodrug modification, which is another effective way to improve the drug action of gemcitabine. NUC-1031 is a gemcitabine phosphoramidate prodrug produced by ProTide technology. The addition of a phosphoramidate motif to gemcitabine can protect it against many of the key cancer resistance mechanisms. Importantly, compared with gemcitabine, NUC-1031 activation was significantly less dependent on dCK and NTs, and it was resistant to cytidine deaminase-mediated degradation [109]. Gemcitabine phosphoramidate prodrug is more active than gemcitabine in the dCK-deficient variants, and its activity against these cell lines is retained in the presence of transport inhibitors. These results are consistent with a mechanism of activation involving intracellular delivery of gemcitabine 5′-monophosphate. Thus, neither deoxycytidine kinase nor the nucleoside transporter is essential for prodrug activity [110]. NUC-1031 is the first anti-cancer ProTide to enter the clinic and has achieved initial success in phase I clinical trials [111]. δ-Tocopherol-monophosphate (MP) gemcitabine (NUC050) is a vitamin E phosphate (VEP)-nucleoside prodrug. VEP-nucleoside prodrugs are designed to address two mechanisms of nucleoside resistance, namely in the case of gemcitabine, which is downregulation of NTs and dCK. Compared to gemcitabine, NUC050 is not affected by NTs inhibitors, suggesting that it can bypass NTs. Similarly, NUC050 retained most of the activity in CEM dCK(−) cells, indicating that NUC050 delivered gemcitabine-MP intracellularly [112].

Recently, the concept of nanoparticle (NP)-based gemcitabine drug delivery has been introduced. By nanoparticle encapsulation and targeted delivery of chemotherapeutic drugs, this encapsulated nanoparticle carrier can easily pass through the cell membrane without being affected by cell surface NTs. It not only overcomes various pathological and pharmacological barriers, but also greatly enhances drug activity and utilization, making it a promising new tool for reducing the chemoresistance of pancreatic cancer. Some researchers developed human serum albumin nanoparticles (GEM-HSA-NP) loaded with gemcitabine. Studies have shown that the nanoparticles loaded with GEM have the ability to inhibit cell proliferation, arrest cell cycle, and induce apoptosis of chemoresistant cancer cells. Most encouragingly, it was equally effective in patients with low expression of hENT1, and further in vivo toxicity evaluation found that the biotoxicity of GEM-HSA-NP was not increased compared with gemcitabine [113]. The nanocarrier method has been further extended to combination therapy [114,115]. A new drug design, which covalently pre-conjugates two or more therapeutic agents via hydrolyzable ligands, makes it possible for various drugs to be loaded onto the same nanocarrier. The paclitaxel–gemcitabine conjugated dual-drug nanocarrier delivery system significantly improved the intracellular efficacy of gemcitabine compared with the free drug conjugates. In many phases II or III clinical trials, the combination of gemcitabine plus nanoparticle bound paclitaxel (nab-paclitaxel) has higher activity and stronger cytotoxicity than GEM alone. Clinical data show that patients with pancreatic cancer receiving gemcitabine combined with nanoparticles have a better survival advantage and higher median OS and PFS [114,115,116,117,118]. A recent meta-analysis compared the effectiveness of GEM plus nab-paclitaxel and FOLFIRINOX in the first-line therapy of advanced pancreatic cancer; median OS in the FOLFIRINOX group was longer compared to GEM-NAB, and the overall risk of death and progression was similar. Side effects were uncommon, with less neurotoxicity and anemia with FOLFIRINOX as compared to less nausea, neutropenia, and febrile neutropenia with GEM-NAB [119]. The modification of gemcitabine to alter its cytotoxicity in cells is attracting more and more attention. Therapeutic regimens, such as gemcitabine plus nab-paclitaxel, have become the first-line choice of chemotherapy for pancreatic cancer patients.

## 7. Genome-Scale Screening Drug Resistant Genes

The main problem with drug treatment for cancer is to validate targets for drugs and related resistance genes. As the drug resistance increases during chemotherapy, the therapeutic effect is gradually reduced, and the identification of new cancer therapeutic targets is crucial for the treatment of drug-resistant cancer. The CRISPR-Cas9 gene editing system has been widely used in cancer treatment and exploration since its discovery. Direct targeting of cancer cell genomic DNA using CRISPR-Cas9 has revealed a wide range of applications and value in the treatment of cancer [120,121,122,123]. Recently, the development and application of CRISPR-Cas9 genome-scale screening methods have made it possible to screen for drug resistance genes [124,125,126,127]. The genome-scale library consists of 64,751 unique sgRNAs and targets 18,080 genes [125]. The sgRNA library can be transfected into cancer cells via lentiviral vectors, and drug-resistant genes can be identified by drug screening and next-generation sequencing technologies [128,129].

A recent study based on genome-scale CRISPR-Cas9 screening aimed to identify factors that modulate NUC-1031 sensitivity [128]. In order to identify genes involved in regulating resistance/sensitivity to NUC-1031 or gemcitabine, the CRISPR-Cas9 genome-scale library was transfected into the MiaPaCa2 cell line via a lentiviral vector. Survival cells were collected 14 and 21 days after treatment with NUC-1031 or gemcitabine. Due to the gene editing role of cas9 and sgRNA, the deletion of certain genes results in partial cell survival after exposure to chemotherapy drugs, and these sgRNAs are better enriched. The remaining sgRNAs can be detected by next-generation sequencing technology, and comparative analysis can screen out key genes that mediate chemoresistance. The results identified and verified that DCK and DCTPP1 are key genes that mediate NUC-1031 resistance [128]. Based on this screening method and theory, new drug resistance genes and anti-drug resistance targets were discovered [130,131]. The methods of gene editing and genome-scale CRISPR-Cas9 knockout screening provide a new direction for exploring drug resistance-related genes and drug-resistance mechanisms.

## 8. Conclusions and Future Directions

As the incidence of pancreatic cancer increases and other cancer prognosis improves, pancreatic cancer will exceed hepatocellular, colon, lung, and prostate cancer by 2030, and is predicted to become the second leading cause of cancer death in western countries [1,2]. The survival rate of pancreatic cancer patients has not presented profound changes in the past 40 years. This grim fact proves the stubbornness of this cancer. One of the main challenges in pancreatic cancer is the solution to its resistance to chemotherapy. Over the past decade, research on the mechanisms underlying drug resistance has contributed to our understanding of this grievous disease. Gemcitabine has become a widely used chemotherapy drug for progressive and metastatic pancreatic cancer since it was first reported [10], although it only has a slight impact on patient survival. Recent phase III clinical studies indicate positive results and helped formulate the new first-line treatment plans of FOLFIRINOX and the doublet of gemcitabine plus nab-paclitaxel [118,119]. A recent phase II clinical trial proved that total neoadjuvant FOLFIRINOX and losartan followed by chemoradiotherapy was related to the margin-negative (R0) resection rate that surpassed expectations in this incurable disease. In total, 42 (86%) patients underwent attempted surgery, with R0 resection achieved in 34 of 49 patients (69%). Overall median PFS was 17.5 months and median OS was 31.4 months. Among the patients who received resection, median PFS was 21.3 months and median OS was 33.0 months [132]. This provides strong evidence for an optimal treatment of pancreatic cancer.

Although progress has been made in the treatment of pancreatic cancer, gemcitabine is still the basis for neo-adjuvant, adjuvant, and palliative therapy for advanced PDAC. Chemoresistance in pancreatic cancer is multifaceted, therefore, pursuing the goal of improving the efficacy of chemotherapy is still challenging. Significant efforts are being made to overcome the limitations inherent in gemcitabine transport mechanisms, activation, and overall clinical response. In order to improve PDAC chemotherapy, the tumor microenvironment and stromal components are promising research directions, which are of great significance for the emergence of chemoresistance mechanisms.

## Figures and Tables

**Table 1:** Potential ways to improve gemcitabine uptake and efficacy.

Method		Target	Mechanism	References
thymidylate synthase inhibitor		hENT1	enhance hENT1 expression, enhance intracellular transport of gemcitabine	[22]
inhibition of MUC4 and its membrane partner		hCNT1 and hCNT3	enhance gemcitabine sensitivity via upregulation of hCNT1 and hCNT3 expression	[101]
prodrug modification	NEO6002, gemcitabine-cardiolipin conjugate	Prodrug, bypass NTs	bypass NTs through drug modification	[103]
	CP-4126, gemcitabine-elaidic acid conjugate	Prodrug, bypass hENT1	transport into cancer cells independent of hENT1 levels	[104,105]
	NUC-1031, NUC050 gemcitabine-phosphoramidate	Prodrug, bypass NTs and dCK	bypass NTs and dCK through prodrug modification	[109,110,111,112]
nanocarrier	GEM-HSA-NP	gemcitabine drug delivery based on NP	overcome various pathological and pharmacological barriers	[113]
	GEM-NAB	gemcitabine drug delivery based on NP	overcome various pathological and pharmacological barriers	[114,115,116,117,118]

Abbreviations: transmembrane glycoprotein mucin 4, MUC4; nucleoside transporters, NTs; nanoparticles, NP; gemcitabine-loaded human serum albumin nanoparticles, GEM-HSA-NP; gemcitabine plus nanoparticle bound paclitaxel (nab-paclitaxel), GEM-NAB.

## Abbreviations

5-FU	5-fluorouracil
CAFs	cancer-related fibroblasts
dCK	deoxycytidine kinase
dCTP	deoxycytidine triphosphate
dFdC	2′,2′-difluoro-2′-deoxycytidine
ECM	extracellular matrix
EMT	epithelial-mesenchymal transition
MSCs	mesenchymal stem cells
NT	nucleoside transporter
OS	overall survival
PDAC	pancreatic ductal adenocarcinoma
PSCs	pancreatic stellate cells
RR	ribonucleotide reductase
TS	thymidylate synthase
